# Epidemiologic, clinical, and serum markers may improve discrimination between bacterial and viral etiologies of childhood pneumonia

**DOI:** 10.3389/fmed.2023.1140100

**Published:** 2023-05-18

**Authors:** Helmia Farida, Rina Triasih, Dewi Lokida, Yan Mardian, Gustiani Salim, Wahyu Nawang Wulan, Deni P. Butar-butar, Rizki Amalia Sari, Arif Budiman, Chakrawati Hayuningsih, Moh Syarofil Anam, Setya Dipayana, Mujahidah Mujahidah, Amalia Setyati, Abu Tholib Aman, Adhella Menur Naysilla, Nurhayati Lukman, Aly Diana, Muhammad Karyana, Ahnika Kline, Aaron Neal, H. Clifford Lane, Herman Kosasih, Chuen-Yen Lau

**Affiliations:** ^1^Rumah Sakit Umum Pusat Dr. Kariadi Hospital/Diponegoro University, Semarang, Indonesia; ^2^Rumah Sakit Umum Pusat Dr. Sardjito Hospital/Universitas Gadjah Mada, Yogyakarta, Indonesia; ^3^Tangerang District General Hospital, Tangerang, Indonesia; ^4^Indonesia Research Partnership on Infectious Disease, Jakarta, Indonesia; ^5^National Institute of Health Research and Development, Ministry of Health Republic of Indonesia, Jakarta, Indonesia; ^6^National Institute of Allergy and Infectious Diseases, Bethesda, MD, United States; ^7^National Cancer Institute, Bethesda, MD, United States

**Keywords:** pediatric, community acquired pneumonia, bacterial, viral, performance characteristics

## Abstract

**Background:**

Discrimination of bacterial and viral etiologies of childhood community-acquired pneumonia (CAP) is often challenging. Unnecessary antibiotic administration exposes patients to undue risks and may engender antimicrobial resistance. This study aimed to develop a prediction model using epidemiological, clinical and laboratory data to differentiate between bacterial and viral CAP.

**Methods:**

Data from 155 children with confirmed bacterial or mixed bacterial and viral infection (*N* = 124) and viral infection (*N* = 31) were derived from a comprehensive assessment of causative pathogens [Partnerships for Enhanced Engagement in Research-Pneumonia in Pediatrics (PEER-PePPeS)] conducted in Indonesia. Epidemiologic, clinical and biomarker profiles (hematology and inflammatory markers) were compared between groups. The area under the receiver operating characteristic curve (AUROC) for varying biomarker levels was used to characterize performance and determine cut-off values for discrimination of bacterial and mixed CAP versus viral CAP. Diagnostic predictors of bacterial and mixed CAP were assessed by multivariate logistic regression.

**Results:**

Diarrhea was more frequently reported in bacterial and mixed CAP, while viral infections more frequently occurred during Indonesia’s rainy season. White blood cell counts (WBC), absolute neutrophil counts (ANC), neutrophil-lymphocyte ratio (NLR), C-reactive protein (CRP), and procalcitonin (PCT) were significantly higher in bacterial and mixed cases. After adjusting for covariates, the following were the most important predictors of bacterial or mixed CAP: rainy season (aOR 0.26; 95% CI 0.08–0.90; *p* = 0.033), CRP ≥5.70 mg/L (aOR 4.71; 95% CI 1.18–18.74; *p* = 0.028), and presence of fever (aOR 5.26; 95% CI 1.07–25.91; *p* = 0.041). The model assessed had a low R-squared (Nagelkerke *R*^2^ = 0.490) but good calibration (*p* = 0.610 for Hosmer Lemeshow test). The combination of CRP and fever had moderate predictive value with sensitivity and specificity of 62.28 and 65.52%, respectively.

**Conclusion:**

Combining clinical and laboratory profiles is potentially valuable for discriminating bacterial and mixed from viral pediatric CAP and may guide antibiotic use. Further studies with a larger sample size should be performed to validate this model.

## Introduction

Community-acquired pneumonia (CAP) is a significant cause of pediatric morbidity and mortality worldwide. The social and economic burden of pediatric CAP is enormous (1). Yet discriminating bacterial and viral etiologies of pneumonia in children remains challenging (2). Despite evidence that appropriate antibiotics are lifesaving, in low-resource settings, rational selection of antibiotics for pneumonia is hampered by limited access to gold standard diagnostic tests, which are often costly and require specialized equipment and techniques (3, 4). Consequently, healthcare providers, particularly those in low-income and middle-income countries (LMIC), likely overtreat non-bacterial pneumonia with antibiotics, which may accelerate development of antimicrobial resistance (AMR) (2, 3, 5). Moreover, prolonged hospital stays and extended medical treatment for antibiotic-resistant infections increase the financial burden (6). Etiologies of CAP have recently been characterized by several studies utilizing advanced molecular testing (4, 7, 8). The notable emergence of viral etiologies may be associated with deployment of *haemophillus influenzae* and pneumococcus vaccines (9–11).

Novel and less expensive strategies for distinguishing between bacterial and viral etiologies of pediatric CAP are needed (6, 12). Alternative predictors of probable bacterial or viral epidemiological agents should be sought when molecular biology tests are unavailable. Many studies have shown that several biomarkers, such as simple blood count and serum biomarkers, including C-reactive protein (CRP) and procalcitonin (PCT), provide clues for differentiating between bacterial and viral infections (12–15). However, the studies found varying utility of those biomarkers (6, 16), likely resulting from small sample sizes, lack of accurate tests for categorizing bacterial and viral pneumonia, and differences in case groups, severity of disease, and comparison groups across studies (15).

The Partnerships for Enhanced Engagement in Research-Pneumonia in Pediatrics (PEER-PePPeS) study was a multisite observational cohort study in Indonesia designed to identify etiologies of childhood CAP using comprehensive diagnostic methods (4). Causative bacterial, viral or mixed viral-bacterial pneumonia pathogens were determined using a previously published algorithm based on microbiological, molecular and serological testing as the gold standard (2). We herein used epidemiologic, clinical and biomarker data for PEER-PePPeS children with known bacterial and mixed versus viral CAP to develop a prediction model using epidemiology, clinical data and common serum biomarkers to improve differentiation between bacterial and mixed versus viral CAP.

## Methods

### Study design and population

Data from children with confirmed bacterial or mixed bacterial and viral CAP were derived from the PEER-PePPPeS study, a prospective cohort study evaluating etiologic agents in hospitalized children with CAP in Indonesia. PEER-PePPPeS was conducted by the Indonesia Research Partnership on Infectious Disease (INA-RESPOND) from July 2017 to September 2019. Sites initially included three government referral teaching hospitals in three provinces: Kariadi Hospital (Central Java), Sardjito Hospital (Yogyakarta), and Tangerang District Hospital (Banten) (4). Satellite sites near the primary sites were added during the study to facilitate subject recruitment. Identification and selection of subjects have been described previously (4). In brief, the study enrolled hospitalized children aged 2–59 months who met the definition of pneumonia within 24 h of admission. Subjects were excluded if they had been hospitalized >24 h; had a malignancy or history of malignancy; had a history of long-term (>2 months) steroid therapy; or had conditions that might interfere with compliance with study procedures.

### Ethics approval

This study was approved by the Ethical Clearance Committee of the Faculty of Medicine, University of Indonesia (No. 567/UN2.F1/ETIK/2017). The study was conducted in accordance with the Declaration of Helsinki. ClinicalTrials.gov Identifier: NCT03366454. Written informed consent was obtained from parents or guardians before enrollment.

### Study procedures

Demographic and clinical data, blood, nasopharyngeal/oropharyngeal (NP/OP) swabs, and induced sputum (IS) were collected at enrollment. As part of standard medical care, blood specimens were tested for routine blood count [white blood cell counts (WBC), absolute neutrophil counts (ANC), and neutrophil-lymphocyte ratio (NLR)], and for inflammatory markers (CRP and PCT). Blood specimens also underwent culture, serology and molecular testing; NP/OP swabs were evaluated by molecular tests; and IS specimens were evaluated by microscopy, culture and molecular tests. On Day 14, we performed clinical examinations and collected convalescent sera. Routine blood count, blood culture, IS culture, Gram stain, CRP, and PCT were performed by the laboratory department at the hospital sites; qPCR, serology and cytokine assays were performed retrospectively at the INA-RESPOND Reference Laboratory located in Tangerang District Hospital.

**Figure 1:**
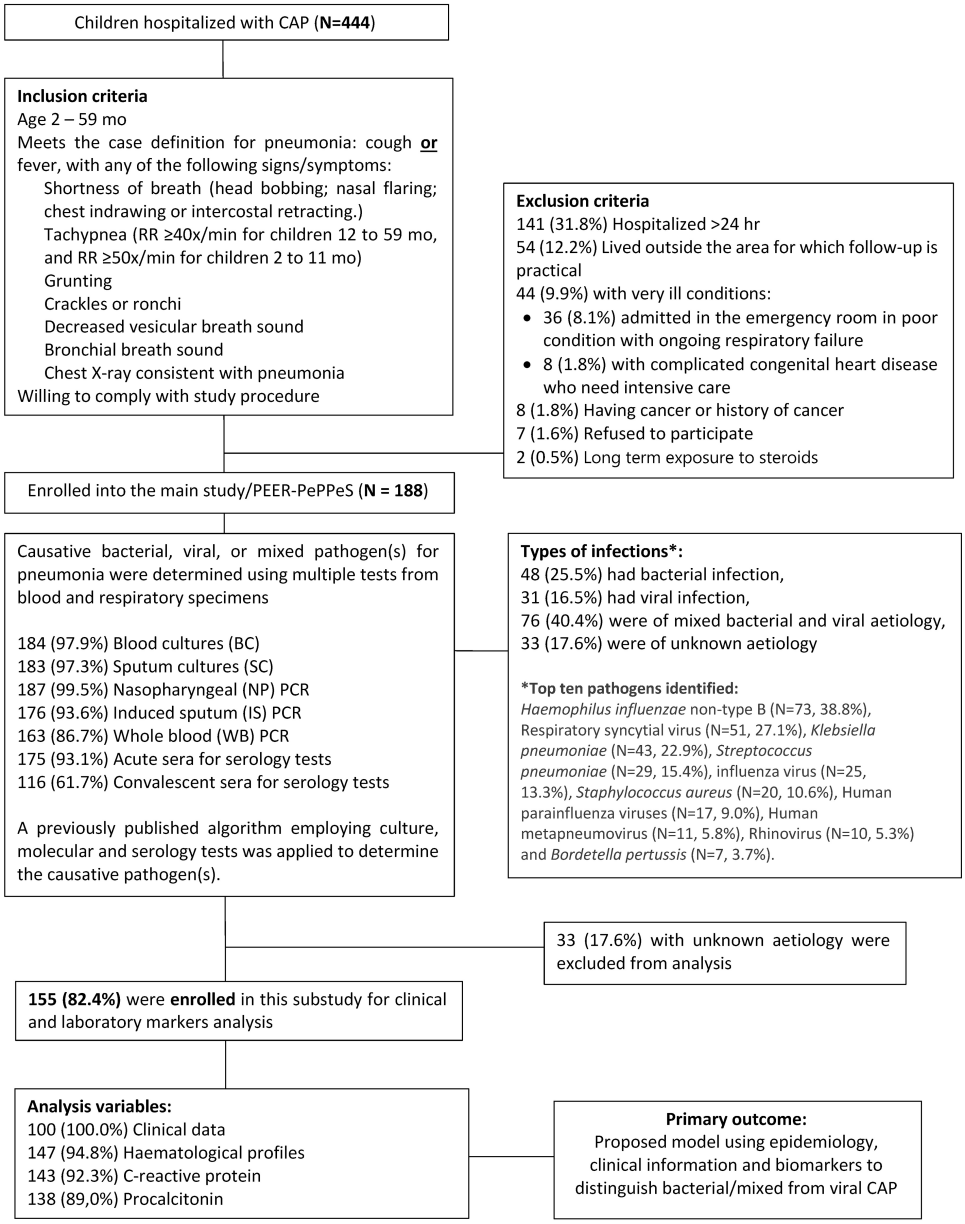
Study flowchart.

PEER-PePPPeS used several widely available bacterial and viral respiratory molecular pathogen panels and serologic assays to determine causative etiologic agents per an algorithm (PEER-PePPeS rules) described previously (2). Based on pathogens identified by the rules, CAP cases were classified as viral, bacterial, or mixed viral-bacterial infection. In this nested study, we included only PEER-PePPeS subjects with known causative agents (*N* = 155) based on the algorithm. Since both bacterial only and mixed viral-bacterial CAP cases would receive antibiotics, we combined the bacterial and mixed viral-bacterial into one group to be compared with the viral group. Epidemiologic, clinical and biomarker profiles were assessed for ability to discriminate bacterial and mixed from viral infections. A study flowchart is shown in Figure 1.

### Measurement of biomarkers

Automated hematology analyzers (Sysmex XN-1000, Sysmex, Kobe, Japan) were used to rapidly analyze whole blood specimens for routine blood count. ANC was determined by multiplying the total WBC count by the percentage of segmented neutrophils plus bands, while NLR was calculated as a simple ratio between the neutrophil and lymphocyte count. CRP assays were performed on the clinical chemistry automated analyzer (ABX PENTRA 400) with a limit detection of 0.10 mg/L. Procalcitonin was measured using the quantitative immunochromatographic assay instrument (the RAMP^®^ Procalcitonin assay) with a limit detection of 0.20 ng/mL.

### Data collection and statistical analysis

Research staff recorded data on paper case report forms and entered them in duplicate into OpenClinica (OpenClinica, LLC, MA, United States). Categorical variables were summarized using absolute values and percentages; continuous variables were described as medians and interquartile ranges (IQRs) for non-parametric data. Differences in epidemiologic, clinical and biomarker data between bacterial/mixed and viral infections were evaluated using bivariate analyses (Pearson χ^2^, Fisher’s exact test, independent-samples *t*-test or the Mann–Whitney U test) as appropriate. The area under the receiver operating curve (AUROC) for varying biomarker levels was used to characterize their utility for discriminating bacterial/mixed and viral CAP and for determining the best cut-off values. These cut-off values were used to classify the biomarkers into categorical variables. The categorical variables with *p* < 0.2 on bivariate analyses were then evaluated by multivariate logistic regression analysis, using the “enter” method to determine most important diagnostic predictors of bacterial/mixed CAP with their adjusted Odds Ratios. Predictor variables with multi-collinearity, assessed by the Variance Inflation Factor (VIF) technique, were excluded from the logistic regression model. Among collinear variables, we chose the highest AUC to be exported to logistic regression. The model’s performance was assessed by the Hosmer Lemeshow test for calibration and the Nagelkerke R^2^ statistic for assessing predictive strength of the logistic regression model. We further explored the combination of significant covariates in the multivariate analysis for their overall sensitivity and specificity for discriminating bacterial/mixed and viral pneumonia. Statistical analyses were performed using Statistical Package for Social Science (SPSS) software version 23 (IBM Corporation, Armonk, NY, United States) and GraphPad Prism 9 (San Diego, CA, United States). A *p*-value (two-tailed) <0.05 was considered statistically significant.

## Results

Among 155 patients prospectively enrolled in the PEER-PePPeS study with a known etiology of CAP, 48 (31.0%) had bacterial infection, 31 (20.0%) had viral infection, and 76 (49.0%) were of mixed bacterial and viral etiology. The top 10 pathogens identified were *Haemophilus influenzae* non-type B (*N* = 73, 38.8%), respiratory syncytial virus (*N* = 51, 27.1%), *Klebsiella pneumoniae* (*N* = 43, 22.9%), *Streptococcus pneumoniae* (*N* = 29, 15.4%), influenza virus (*N* = 25, 13.3%), *Staphylococcus aureus* (*N* = 20, 10.6%), human parainfluenza viruses (*N* = 17, 9.0%), human metapneumovirus (*N* = 11, 5.8%), rhinovirus (*N* = 10, 5.3%), and *Bordetella pertussis* (*N* = 7, 3.7%) (Figure 1). The etiological characteristics, pathogen distribution between age groups and study sites, mortality and seasonality pattern of this cohort have been presented previously (4).

### Demographic, clinical, and laboratory characteristics

A total of 155 children with confirmed bacterial or mixed infection (*N* = 124) and viral infection (*N* = 31) were included in this nested study. Table 1 shows the demographic, clinical and laboratory characteristics of the study population. Diarrhea was more frequently reported in bacterial and mixed CAP, while viral infections occurred more frequently during the wet season in Indonesia. WBC count, ANC, NLR, CRP, and PCT were significantly higher in bacterial and mixed cases. There was no difference in other baseline characteristics, including age, gender, comorbidities, pneumonia severity, X-ray findings, mortality, and complications between these two groups.

**Table 1:** Demographic, clinical, and laboratory characteristics.

Parameter	Bacterial and mixed infection (*N* = 124)	Viral infection (*N* = 31)	*p*-value
Age, median (IQR) months	9.0 (5.0–19.0)	13.0 (5.5–22.5)	0.211^mw^
Gender, male, (%)	72 (58.1)	13 (41.9)	0.107^cs^
Household characteristics, (%)			
Living in a dense neighborhood*	81 (65.3)	22 (71.0)	0.552^cs^
Living near waste disposal	43 (34.7)	14 (45.2)	0.279^cs^
Sick household contact <14 days	67 (54.0)	20 (64.5)	0.293^cs^
Firewood cooking	15 (12.1)	4 (12.9)	0.903^cs^
Attending day-care facilities	3 (2.5)	0 (0)	1.000^f^
Infection occurred during rainy season in Indonesia (November to March), (%)	49 (39.5)	19 (61.3)	**0.029**^**cs**^
Comorbidities, (%)			
Congenital heart disease	30 (24.6)	8 (25.8)	0.889^cs^
Severe malnutrition^†^	19 (15.3)	5 (16.1)	1.000^f^
Developmental delay	29 (24.2)	10 (32.2)	0.359^cs^
Neurologic disorder	12 (9.7)	6 (19.4)	0.205^f^
Asthma	3 (2.4)	3 (9.7)	0.098^f^
Allergy	6 (4.8)	2 (6.5)	0.662^f^
Premature birth	23 (18.5)	6 (19.4)	0.918^cs^
Low birth weight	31 (25.0)	8 (25.8)	0.945^cs^
Symptoms and signs at baseline (%)			
Cough	116 (93.5)	27 (87.1)	0.260^f^
Shortness of breath	116 (93.5)	28 (90.3)	0.461^f^
Fever	105 (84.7)	22 (71.0)	0.076^cs^
Decreased consciousness	6 (4.8)	0 (0)	0.600^f^
Inability to drink	10 (8.1)	2 (6.4)	1.000^f^
Diarrhea^‡^	30 (24.2)	1 (3.2)	**0.009**^**cs**^
Vomiting	9 (7.3)	2 (6.4)	1.000^f^
Seizure	5 (4.0)	1 (3.2)	1.000^f^
Tachypnea	54 (43.5)	13 (41.9)	0.871^cs^
Intercostal retraction	112 (90.3)	27 (87.1)	0.528^f^
Rhonchi	115 (92.7)	25 (80.6)	0.081^f^
Wheezing	20 (16.1)	2 (6.4)	0.251^f^
Nasal flaring	49 (39.5)	11 (35.5)	0.680^cs^
Chest indrawing	85 (68.5)	18 (58.1)	0.269^cs^
Head bobbing	7 (5.6)	2 (6.5)	1.000 ^f^
Skin rash	6 (4.0)	2 (6.5)	0.628 ^f^
SpO_2_ < 90% and/or cyanosis	29 (23.4)	6 (19.4)	0.631^cs^
Laboratory parameter at baseline, median (IQR)			
White Blood Cells (WBC) count, ×10^3^/μL	15.2 (10.8–20.3)	11.5 (8.9–14.0)	**0.005**^**mw**^
Absolute Neutrophil Count (ANC), ×10^3^/μL	9.0 (5.1–13.3)	5.6 (4.1–7.3)	**0.003**^**mw**^
Neutrophil-lymphocyte ratio (NLR)	2.0 (1.0–3.0)	1.2 (0.8–1.7)	**0.023**^**mw**^
C-Reactive Protein (CRP), mg/L	14.1 (4.9–43.9)	4.9 (2.7–11.9)	**0.002**^**mw**^
Procalcitonin (PCT), ng/mL	0.4 (0.1–2.4)	0.1 (0.04–0.2)	**0.001**^**mw**^
Severe pneumonia or very severe disease (WHO classification 2014 version) (%)	55 (44.4)	16 (51.6)	0.468^cs^
Chest X-ray findings (%)			
Pleural effusion	3 (2.4)	1 (3.2)	1.000^f^
Interstitial infiltrate	93 (75.0)	19 (61.3)	0.110^cs^
Alveolar infiltrate	86 (69.4)	19 (61.3)	0.357^cs^
Duration of hospital stay, median (IQR) days	6.0 (4.0–8.0)	5.0 (4.0–10.5)	0.800^mw^
ICU admission (%)	15 (12.1)	3 (9.7)	1.000^f^
Invasive mechanical ventilation (%)	6 (4.8)	2 (6.5)	0.345^f^
Sepsis complication^§^ (%)	9 (7.3)	1 (3.2)	0.688^f^
In-hospital mortality (%)	11 (8.9)	2 (6.5)	1.000^f^

*A densely populated neighborhood was defined as >200 people/km^2^ or < 8 m^2^/person in the subject’s home. ^†^Severe malnutrition was defined as weight for height below −3 SD from the median of the WHO Child Growth Standards. ^‡^Diarrhea was defined by the passage of 3 or more loose or liquid stools per day or more frequently than is normal for the individual. ^§^Sepsis complication was defined as pneumonia complicated by sepsis during hospitalization. ^mw^Mann–Whitney U test; ^cs^Pearson chi-square test; ^f^Fisher’s exact test. Bold values indicate statistically significant at the 0.05 (or 5%) level.

### Predictive accuracy of single biomarkers for distinguishing bacterial and viral CAP

**Figure 2:**
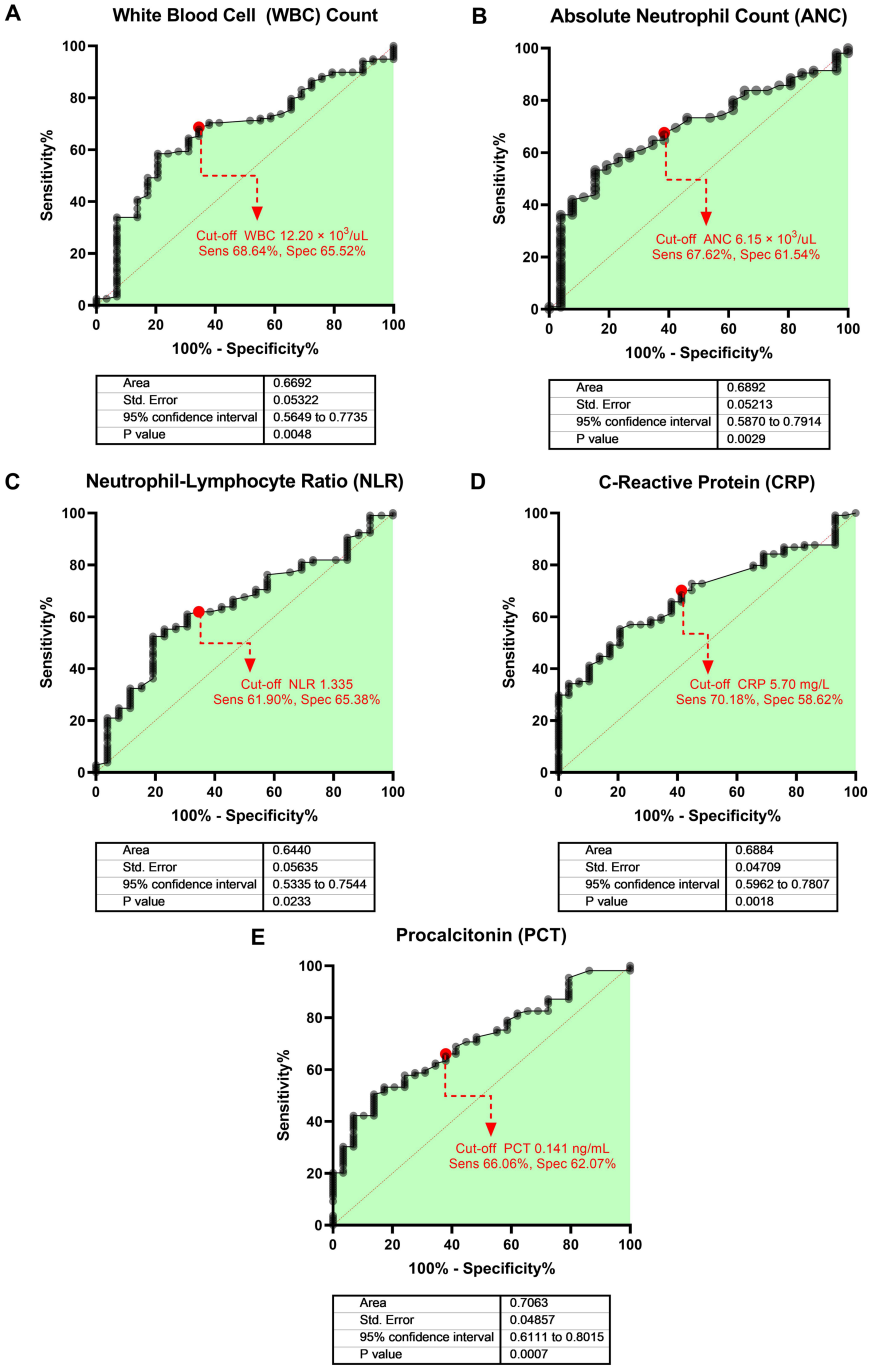
Area under the receiver operating characteristic (AUROC) of each biomarker for evaluating the discriminative power of bacterial and mixed infection or viral infection. The bold heading for each paned indicates the biomarker evaluated: **(A)** WBC count, **(B)** ANC, **(C)** NLR, **(D)** CRP, and **(E)** PCT. The dots with dashed lines represent the best cut-off values based on the Youden index.

Among single biomarkers, PCT showed the best discriminatory ability (AUROC 0.71, 95% CI 0.61–0.80, *p* = 0.001), followed by CRP (AUROC 0.69, 95% CI 0.60–0.78, *p* = 0.002), and ANC (AUROC 0.69, 95% CI 0.59–0.79, *p* = 0.003) (Figure 2). Table 2 shows the sensitivity, specificity, and predictive values of selected cut-off points of each biomarker that showed significant discriminatory ability on ROC analysis. We determined cut-off points, based on the highest Youden index (sensitivity + specificity). For example, a cut-off score of PCT ≥0.141 ng/mL showed the highest Youden index among other cut-off values with moderate sensitivity (66.06%) and specificity (62.07%).

**Table 2:** Diagnostic performance of biomarker cut-off scores for bacterial and mixed viral and bacterial infections.

Biomarker	Cut-off score	Sensitivity (%)	Specificity (%)	Positive predictive value (%)	Negative predictive value (%)
WBC	≥12.20 × 10^3^/μL	68.64	65.52	89.01	33.93
ANC	≥6.15 × 10^3^/μL	67.62	61.54	87.65	32.00
NLR	≥1.335	61.90	65.38	87.84	29.82
CRP	≥5.70 mg/L	70.18	58.62	86.96	33.33
PCT	≥0.141 ng/mL	66.06	62.07	86.75	32.73
CRP + fever	CRP ≥ 5.70 mg/L	62.28	65.52	87.65	30.64
CRP + dry season	CRP ≥ 5.70 mg/L	40.35	82.76	90.19	26.09
CRP + fever + dry season	CRP ≥ 5.70 mg/L	35.09	86.21	90.91	25.25

### Logistic regression analyses

Categorical variables with value of *p* < 0.2 on bivariate analyses were evaluated by the logistic regression model (Table 3). For biomarkers, we first tested the multicollinearity of the data using the Variance Inflation Factor (VIF) technique, and found that WBC count, NLR and ANC showed high collinearity (VIF >2, data not shown), which was expected since their values were related each other. Of the three, ANC was selected for inclusion in the logistic regression model because it had the highest AUROC. Based on each biomarker’s predetermined cut-off (Table 2), we then dichotomized those variables for logistic regression analysis and selected those for which the bivariate analysis, incorporating the binary epidemiological and clinical characteristics data, showed *p* < 0.2.

**Table 3:** Univariate and multivariate logistic regression model to predict bacterial or mixed infection.

Variables/covariates	Univariate analysis		Multivariate analysis*	
	Crude OR (95%CI)	*p*-value	Adjusted OR (95% CI)	*p*-value
Age ≤ 1 year-old	1.69 (0.77–3.73)	0.192		
Male	1.92 (0.86–4.26)	0.107		
Asthma comorbidity	0.24 (0.05–1.23)	0.098		
Fever	2.26 (0.90–5.65)	0.076	5.26 (1.07–25.91)	0.041
Ronchi	3.07 (1.00–9.40)	0.042		
Diarrhea	9.57 (1.25–73.22)	0.009		
Rainy season	0.41 (0.18–0.93)	0.029	0.26 (0.08–0.90)	0.033
Interstitial infiltrate on CXR	1.96 (0.85–4.50)	0.110		
ANC ≥ 6.15 × 10^3^/μL	3.34 (1.37–8.13)	0.006		
CRP ≥ 5.70 mg/L	3.33 (1.44–7.73)	0.004	4.71 (1.18–18.74)	0.028
PCT ≥ 0.141 ng/mL	3.18 (1.36–7.44)	0.006		

*Logistic regression Nagelkerke R square = 0.490, Hosmer Lemeshow test *p* = 0.610.

All variables with a value of *p* < 0.2 on univariate analysis were evaluated by multivariate analysis. The following variables were found to be the most important predictors of bacterial or mixed CAP: rainy season (aOR 0.26; 95% CI 0.08–0.90; *p* = 0.033), CRP ≥ 5.70 mg/L (aOR 4.71; 95% CI 1.18–18.74; *p* = 0.028), and presence of fever (aOR 5.26; 95% CI 1.07–25.91; *p* = 0.041) (Table 3). The model assessed had good calibration (*p*-value 0.610 for Hosmer Lemeshow test) and low predictive strength (Nagelkerke *R*^2^ = 0.490). We further explored the combination of the most important predictors, and found that the combination of CRP ≥ 5.70 mg/L and the presence of fever resulted in moderate predictive value with sensitivity and specificity of 62.28 and 65.52%, respectively (Table 2).

## Discussion

In this nested study, we established a model combining epidemiologic, clinical and biomarker data for predicting bacterial and mixed CAP among pediatric patients enrolled in the PEER-PePPeS study. These predictive variables are routinely available in limited resourse settings, where high yield cultures and molecular assays may be inaccessible. Of note, WBC, NLR, ANC, CRP, and PCT were significantly higher in bacterial and mixed infection groups, compared with the viral-only group. Our model showed that occurrence of infection during the rainy season, CRP ≥5.70 mg/L and presence of fever might inform clinical decision-making for hospitalized pediatric CAP, particularly with regard to antibiotic stewardship. However, the best combination of important predictors (CRP and fever) only provided moderate predictive value with sensitivity and specificity of 62.28 and 65.52%, respectively, thus should be interpreted with caution. Socio-epidemiologic factors were not significant predictors of pediatric CAP etiology in our study, but have been shown by others to correlate with severity of disease (17). We found that objectively measured parameters, such as biomarkers, were most valuable in predicting etiology.

Consistent with previous literature, our study suggests that conventional host biomarkers (WBC count, ANC, CRP, and PCT) provide clues for differentiating between bacterial and viral infections in children with CAP (18). The relationships between acute bacterial infection, high WBC, and high ANC have been recognized for many years due to high availability in acute care settings (19). Slight variation in normal WBC and ANC values by race or ethnicity, such as in persons of African ancestry (20), may influence the relationship. The diagnostic value of WBC count and ANC as indicators of acute bacterial infection, including bacterial pneumonia, remains under investigation (13, 19). In our study, ANC had moderate ability to distinguish bacterial and viral infection per AUROC analysis, but failed to show significance in the multivariate model (Figure 2 and Table 3).

CRP and PCT are the most frequently endorsed biomarkers for identifying bacterial infections in children because their levels are higher in bacterial infections than in viral infections (6). CRP, an acute phase plasma protein, is synthesized by hepatocytes and adipocytes in response to inflammatory cytokines and is widely known as an indicator of acute inflammation (15, 21). CRP was first discovered in sera from pneumonia patients in 1930, when it was shown to precipitate the C-polysaccharide of *Streptococcus pneumoniae* (21, 22). Since then, CRP has been linked to bacterial infections generally, as well as other etiologies of inflammation (21). These associations have led to the use of CRP for discriminating between bacterial and non-bacterial pneumonia (6). While some studies have demonstrated greater CRP levels in bacterial than in viral pneumonia (6, 15), others have not (23, 24). A recent study by Higdon et al. showed that CRP ≥40 mg/L has good specificity for ruling out most RSV pneumonias (83% of RSV pneumonia cases had CRP <40 mg/L). However, approximately 23% of cases with confirmed severe or very severe bacterial pneumonia also had CRP <40 mg/L (15). Thus CRP alone is insufficient for diagnosing bacterial pneumonia. Bhuiyan et al. found that a CRP threshold of 72 mg/L corresponded to an AUROC of 0.82 for discriminating definite bacterial pneumonia from presumed viral pneumonia (25). The CRP cut-off value in our predictive model is different than that of Higdon et al. and Bhuiyan et al. (Tables 2, 3), suggesting that CRP response could vary across populations or that values differ by assay, making it challenging to select a universal cut-off threshold for diagnostic use (15, 26). Of note, the Higdon and Bhuiyan studies were conducted in high-income settings where the contribution of viral infection was higher and could confound the analysis (15, 25). Advantages of CRP compared to PCT include availability of rapid point-of-care tests and low cost (18).

PCT, a protein which consists of 116 amino acids, is the peptide precursor of calcitonin (27, 28). Under normal circumstances, PCT circulates at low concentrations (≤0.1 ng/mL) (27, 28). Among traditional biomarkers, PCT has been shown to be the most effective in identifying bacterial cases. However, a precise cut-off level separating bacterial from viral cases has not been defined (29). PCT was first described in 1993 as a marker of bacterial infection when high concentrations of a calcitonin-like substance were detected in the blood of patients with extra thyroid diseases; serum PCT levels were very high in patients with severe invasive bacterial infections and levels decreased rapidly during antibiotic therapy (27, 30). Subsequently, PCT was shown to be increased during bacterial sepsis and correlate with severity of microbial invasion (6). Our AUROC results align with previous studies suggesting PCT performs better than other biomarkers for distinguishing bacterial pneumonia (14, 18, 31). However, results across studies have been inconsistent (28), undermining the validity of PCT as a solitary predictor (12). Compared to CRP, the major advantage of PCT is its specificity in differentiating bacterial infection from inflammatory syndromes due to noninfectious causes, which could also affect biomarker values observed in our study (32). Universal PCT cut-off values may also be challenging to establish since thresholds were different across studies and age groups (28).

Our multivariate analysis indicates that epidemiologic and clinical data may be useful in distinguishing bacterial/mixed from viral infection. The presence of fever was shown to be important in predicting bacterial/mixed infection, consistent with previous studies which have shown that bacterial infection is more frequently associated with high-grade fever than viral pneumonia (25, 33). The combination of CRP and presence of fever had moderate predictive value; sensitivity and specificity were 62.28 and 65.52%, respectively. Our analysis also showed that pathogen type is associated with season (Table 3). This aligns with previous studies that suggest common respiratory viruses, such as RSV and Influenza virus, are often detected during Indonesia’s wet or rainy season (4, 34, 35). Occurrence of diarrhea more commonly in the bacterial/mixed infections group of our cohort might be explained by systemic bacterial infections being more likely to induce diarrhea (36) or occurrence of antibiotic-associated diarrhea related to antibiotic treatment before hospital admission, although the latter is less likely because subjects were enrolled within 24 h of admission (37). Care should be taken in interpreting this data as they are based on parent/guardian report.

This nested study has several limitations. First, the relatively small sample size limits generalizability of the results. Second, most subjects (79.8%) in PEER-PePPeS received antibiotics before the first specimen collection (4), which might have impacted disease progression, including markers assessed in this study (25). To address this possibility, we enrolled subjects within 24 h of admission, and specimens were collected as soon as possible to minimize the effects of antibiotics on culture results. Third, this study was conducted before identification of COVID-19 in Indonesia (38); thus, the proposed prediction model did not include the role of SARS-CoV-2, and further investigations are warranted. Fourth, our study did not assess the potential utility of some novel biomarkers, such as Myxoma resistance protein (MxA1), High mobility group box one protein (HMGB1), or Tumor necrosis factor-related apoptosis-inducing ligand (TRAIL), which may have better diagnostic performance for distinguishing bacterial and viral infection than conventional biomarkers (39, 40). However, availability for testing these novel biomarkers is scarce in Indonesia, and would thus be less relevant than those we assessed.

In conclusion, our multivariate model is potentially valuable for discriminating bacterial from viral infections and informing antibiotic treatment decisions for pediatric CAP. Our analysis showed that season, presence of fever, and a certain level of CRP can help differentiate bacterial/mixed from viral CAP. However, combining those factors only resulted in moderate sensitivity and specificity. Further studies with a larger sample size in a similar setting (a low-income country with predominant bacterial/mixed infections) should be performed to validate and optimize this model.

## Data availability statement

The raw data supporting the conclusions of this article will be made available by the authors, without undue reservation.

## Ethics statement

The studies involving human participants were reviewed and approved by the Ethical Clearance Committee of the Faculty of Medicine, University of Indonesia (No. 567/UN2.F1/ETIK/2017). Written informed consent to participate in this study was provided by the participants’ legal guardian/next of kin.

## Author contributions

HF, RT, DL, YM, HK, and C-YL designed and conceptualized the study. HF, RT, DL, AB, MA, SD, MM, AS, and AA performed clinical assessments and were responsible for data entry. DL, CH, GS, WW, DB-b, and RS performed laboratory assays. YM performed data analysis and interpretation and drafted the first manuscript. DL, AN, AK, HK, and C-YL assisted with manuscript writing, analysis, and interpretation of data. All authors contributed to manuscript development, edited for critical content, and have approved the final version.

## Funding

This manuscript has been funded in whole or in part with MoH Indonesia, National Academy of Sciences (Sub-Grant No. 2000007599), and Federal funds from the NIAID, NIH (Contract Nos. HHSN261200800001E and HHSN261201500003I). The content of this publication does not necessarily reflect the views or policies of the Department of Health and Human Services, nor does mention of trade names, commercial products, or organizations imply endorsement by the U.S. Government.

## Conflict of interest

The authors declare that the research was conducted in the absence of any commercial or financial relationships that could be construed as a potential conflict of interest.

## Publisher’s note

All claims expressed in this article are solely those of the authors and do not necessarily represent those of their affiliated organizations, or those of the publisher, the editors and the reviewers. Any product that may be evaluated in this article, or claim that may be made by its manufacturer, is not guaranteed or endorsed by the publisher.
